# Speech-Based Surgical Phase Recognition for Non-Intrusive Surgical Skills’ Assessment in Educational Contexts

**DOI:** 10.3390/s21041330

**Published:** 2021-02-13

**Authors:** Carmen Guzmán-García, Marcos Gómez-Tome, Patricia Sánchez-González, Ignacio Oropesa, Enrique J. Gómez

**Affiliations:** 1 Biomedical Engineering and Telemedicine Centre, ETSI Telecomunicación, Centre for Biomedical Technology, Universidad Politécnica de Madrid, Madrid 28040, Spain; marcos.gomez.tome@alumnos.upm.es (M.G.-T.); p.sanchez@upm.es (P.S.-G.); i.oropesa@upm.es (I.O.); enriquejavier.gomez@upm.es (E.J.G.); 2 Centro de Investigación Biomédica en Red en Bioingeniería, Biomateriales y Nanomedicina, Madrid 28029, Spain

**Keywords:** natural language processing, surgical workflow analysis, speech analysis, machine learning, feature extraction, surgical process model

## Abstract

Surgeons’ procedural skills and intraoperative decision making are key elements of clinical practice. However, the objective assessment of these skills remains a challenge to this day. Surgical workflow analysis (SWA) is emerging as a powerful tool to solve this issue in surgical educational environments in real time. Typically, SWA makes use of video signals to automatically identify the surgical phase. We hypothesize that the analysis of surgeons’ speech using natural language processing (NLP) can provide deeper insight into the surgical decision-making processes. As a preliminary step, this study proposes to use audio signals registered in the educational operating room (OR) to classify the phases of a laparoscopic cholecystectomy (LC). To do this, we firstly created a database with the transcriptions of audio recorded in surgical educational environments and their corresponding phase. Secondly, we compared the performance of four feature extraction techniques and four machine learning models to find the most appropriate model for phase recognition. The best resulting model was a support vector machine (SVM) coupled to a hidden-Markov model (HMM), trained with features obtained with Word2Vec (82.95% average accuracy). The analysis of this model’s confusion matrix shows that some phrases are misplaced due to the similarity in the words used. The study of the model’s temporal component suggests that further attention should be paid to accurately detect surgeons’ normal conversation. This study proves that speech-based classification of LC phases can be effectively achieved. This lays the foundation for the use of audio signals for SWA, to create a framework of LC to be used in surgical training, especially for the training and assessment of procedural and decision-making skills (e.g., to assess residents’ procedural knowledge and their ability to react to adverse situations).

## 1. Introduction

### 1.1. Clinical Context

Surgery has been categorized as one of the most psychologically demanding professions [1]. This is because surgeons must execute complex technical procedures while satisfying cognitive demands such as decision making, clinical judgment or problem solving. Thus, many educational solutions have been proposed to support residents’ learning processes [2,3], for instance by contributing to the objective assessment of technical and non-technical skills [4].

Surgeons’ procedural and intraoperative decision-making skills are essential for clinical practice [5]. Procedural skills refer to the in-depth knowledge of the surgical steps in which a procedure is divided, while decision-making skills are defined as the ability to choose the best course of action after carefully analyzing and examining the available options [6]. Residents and educators alike are concerned about the objective assessment of these skills and have been looking at strategies to address this issue [7].

Uemura et al. suggested that modelling surgical processes could allow to implement an assessment methodology for developing the procedural and decision-making skills necessary when facing complications in the operating room (OR) [4]. Surgical process modelling (SPM) has been defined by Lalys and Jannin as “simplified patterns of surgical processes that reflect a predefined subset of interest of the surgical process in a formal or semi-formal representation” [8]. SPM analyzes different information sources in the OR to determine the state of the surgical process. These sources may be monitored using different methods [8]. For instance, sensors based on RFID technologies have been used for instrument, medications and surgical staff tracking [9,10]. More complex tracking systems have been studied to be applied for the creation of surgical process models, such as eye tracking [11] or ultrasonic systems [12]. Currently, video-based monitoring is under research [13].

One of the steps involved in SPM is surgical workflow analysis (SWA), which is the deconstructing process by which a surgical process is divided into a list of different phases, activities or tasks [9]. This analysis is not straightforward, mainly because it is necessarily different for each surgical procedure. Hence, the methods used for SWA are designed with focus on a particular procedure to optimize the results [8]. Next, we provide a state of the art on the current methods used to perform SWA.

### 1.2. State of the Art

SWA focuses on analyzing video frames to recognize surgical phases and actions. Probabilistic methods have proven to be of great interest in video-based SWA. For instance, Padoy et al. proposed a method based on Hidden-Markov models (HMM) to analyze the presence of surgical instruments and surgical clips on the endoscopic image, as well as information on the location of the camera (i.e., inside or outside the body) [10]. The major challenge they encountered was the interrupted and delayed availability of instruments’ signals. Despite this, they obtained an average accuracy of 93% within 11 surgical procedures. Another probabilistic approach was carried out by Dergachyova et al. [11]. Their model sought to recover the sequence of phases based on low-level visual cues and manually-annotated instrument information. They reached an accuracy of 88.93% when using a combination of both sources.

Probabilistic methods are usually trained with simple features based on low-level visual cues, typically obtained through a manual annotation process, which is virtually impossible to perform for real-time applications [12]. Thus, recent video-based SWA studies have shifted towards the recognition of surgical phases using a combination of deep learning techniques and probabilistic methods (mostly with HMM) [12,13,14,15]. For instance, Twinanda et al. used a convolutional neural network (CNN, EndoNet) to learn features from the images in a laparoscopic cholecystectomy (i.e., removal of the gallbladder) [12]. These features were then passed to a support vector machine (SVM) coupled to an HMM to carry out the phase recognition and tool presence detection tasks. They achieved accuracies ranging from 76.3% to 92.2%.

In order to include temporal information within the feature extraction network, Lea et al. used spatiotemporal convolutional neural networks (STCNN), which essentially capture object motion over short time intervals to extract characteristics from the laparoscopic videos [16]. These features were fed to a time-invariant model based on dynamic time warping (DTW) inspired by Padoy et al. [17]. Results achieved an 85% accuracy.

Following this line of research, Jin et al. created a framework (EndoRCN) consisting of a very deep residual convolutional neural network (RCNN) and a long short-term memory (LSTM) unit [18]. LSTM units are able to train video recognition models that capture temporal state dependencies [19]. This resulted on a 78.2 Jaccard index, which is defined as the size of the intersection divided by the size of the union of two sets (i.e., the predicted phases returned by the classifier and the actual phases). A similar approach was followed by Nakawala et al. [15] and Yengera et al. [16]. Nakawala et al. created a pipeline consisting of an RCNN model made from a CNN coupled with an LSTM unit, and a sequence model made from three additional LSTM units, which was not trained end-to-end. It resulted in an accuracy of 74.29% in the recognition of phases. Yengera et al. trained an end-to-end CNN coupled with one LSTM unit and achieved an accuracy of 86.7%, observing that end-to-end networks boost surgical phase recognition performance.

Shi et al. proposed a non-local recurrent convolutional network (NL-RCNet), which introduces non-local blocks to capture the long-range temporal dependency (LRTD) among continuous frames [20]. LRTD can indicate the cross-frame dependencies among all the frames in a clip, without the limitation of time intervals. More specifically, they integrated a 50-layer residual network (ResNet50) followed by an LSTM unit to form an end-to-end RCNN, to extract features. Based on these features, they employed the non-local block to capture LRTD of frames within each clip, yielding an 85.73% accuracy.

Video-based SWA methods are not without limitations. For instance, they need a thorough image processing and an in-depth knowledge of computer vision techniques to yield accurate results [21]. Additionally, videos are particularly prone to errors due to camera occlusions, which are common in the OR [22]. Many perturbations occur (e.g., appearance of smoke, tissue deformation, fast change of field of view…), which pose special challenges to computer vision algorithms [10].

A novel possibility for SWA currently under research considers monitoring audio cues during surgical procedures from the attending surgical staff. To this day, however, the research for speech-based SWA is in its early stages, which creates great scope for the study of the potential of this approach. To our knowledge, the only study in this field was reported by Suzuki et al., which attempted to use audio information to find patterns of conversation (i.e., correlating the amount of conversation to the probability of transitioning from one phase of the procedure to another) [23]. However, their recognition algorithm reached only a 51.6% accuracy.

### 1.3. A New Paradigm for Surgical Workflow Analysis

This study is part of a broader research effort to develop new non-intrusive methods for monitoring skills in surgical educational environments. These environments usually involve a training scenario within the OR, in which surgical models (e.g., virtual simulations, animal models, etc.) are the matter of study instead of real human patients.

**Figure 1:**
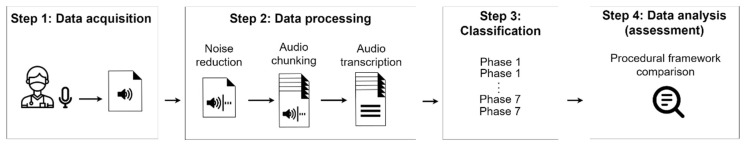
Workflow of the proposed paradigm to aid in the assessment of surgical non-technical skills.

More specifically, we aim to implement a non-intrusive system to aid in the assessment of surgical procedural and decision-making skills within these educational environments (Figure 1). We propose to go one step further in speech-based SWA and analyze the speech of the surgeon-in-training while an educational surgical procedure is taking place to determine the ongoing phase. In this sense, our hypothesis is that speech analysis in surgical educational contexts could provide information of the cognitive processes related to decision making [24,25].

Speech-based SWA requires employing Natural Language Processing (NLP) techniques. NLP is a field of computer science, artificial intelligence and computational linguistics concerned with programming computers to fruitfully process a large amount of human (natural) language data [26]. This includes the conversion from audio signals to text and their further categorization (in our particular case, into the surgical phase in which the surgeon is while speaking). Furthermore, NLP goes one step further and enables machines to infer meaning and sentiment from unstructured data, such as electronic medical records (EMRs) [27]. In fact, many EMRs now incorporate NLP to improve the workflow (i.e., to automatically detect adverse events and postoperative complications) [28,29,30], which proves the effectiveness of NLP’s applications in medical areas.

The system we propose will gather in real time the surgeon-in-training’s voice by means of a clip-on microphone. Trainees will be specifically asked to verbally disclose each of the actions they are performing to be analyzed within the framework of the procedure. This audio will be processed to reduce the noise in the OR, chunked into smaller segments and transcribed into text by an automatic transcription tool. The resulting text will be analyzed by a classification algorithm and the phases predicted will be compared to a framework of the procedure’s phases and actions as a support for the assessment of surgical procedural and decision-making skills.

Ultimately this system could also serve as a training tool to guide surgical residents in the procedure, alerting them in real time of missed steps and suggesting different pathways. Such system may even be upgraded with a dictionary of possible solutions to complications encountered.

### 1.4. Goals of This Study

This study focuses on the implementation of the third step of the above-presented paradigm. Specifically, the goal of this study is to research different machine-learning algorithms for speech-based SWA. We present a comparison between different NLP-based feature extraction techniques and classification models to classify the phases of a surgical procedure using audio signals. The results of this study will ultimately provide insights on the feasibility of a framework for speech-based SWA as a means to characterize the cognitive processes behind surgical decision making.

## 2. Materials and Methods

**Figure 2:**
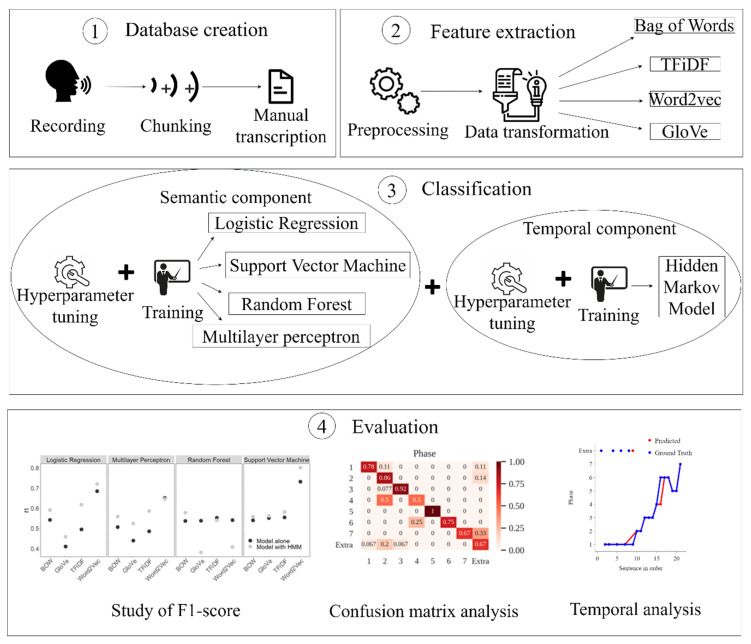
Pipeline of the methods carried out in this study.

The overall pipeline process of the proposed algorithm is presented in Figure 2. The first step consists of the creation of a database using the manual transcriptions of audio files recorded in surgical educational environments. The second step consists of the extraction of features from the obtained transcriptions by means of four different feature extraction techniques. The final step consists of the training and the evaluation of four different machine learning models coupled with an HMM to capture the temporal information of the surgical process.

### 2.1. Database Creation

Laparoscopic cholecystectomy (LC) was selected as a study case for this work. LC involves the removal of the gallbladder necessitated by the formation of gallstones or polyps. This interventional technique is one of the most widespread and convenient procedures for SWA (due to the unique characteristics of each of its phases), as well as one of the interventions with the highest training rate [12].

To create the database with which to train and test the classification of LC phases, we selected 15 online surgical educational videos, in which the steps of the educational surgical procedure were verbally disclosed by a single trainer surgeon equipped with a clip-on microphone. The selection criteria were that the videos (1) were verbally commented in Spanish, (2) detailed the steps followed during the procedure, (3) explicitly mentioned the anatomic landmarks shown in camera and (4) identified the complications encountered during the procedure while verbally mentioning how to solve them.

The audio from the videos was extracted and chunked into smaller segments according to the silences present in the recording, using Python’s speech_recognition module [31]. This allowed to have smaller audio sequences which could be easily associated to a specific LC phase. For this study, we divided LC into 8 phases. The first 7 phases are compatible with the phases defined in the database “Cholec80” [12], provided by the University of Strasbourg: (1) preparation, (2) Calot’s triangle dissection, (3) cystic duct and artery clipping and cutting, (4) gallbladder dissection, (5) gallbladder extraction, (6) cleaning and coagulating, (7) suturing and port removal (Table 1). An “extra” pseudo-phase was defined to account for audio not necessarily related to the procedure itself (i.e., normal conversation between surgeons during the educational procedure).

Each of the obtained audio segments was manually transcribed into text and assigned to one of these phases independently by two different researchers with background in the area (inter-annotator correlation, Cohen-Kappa (к) = 0.926). Both transcriptions and phases were pooled and verified.

The resulting transcribed text formed the database to be used in this study (Table 2). In total, 611 text samples (i.e., transcriptions of audio segments) were obtained and labelled. A total of two full-length videos (with 58 samples in total) were set aside to test the final model. The remaining 13 videos (553 samples) were used as part of the training set.

### 2.2. Feature Extraction

NLP commonly follows a two-step pipeline [32]. The first step (data pre-processing) generally consists of (1) a normalization of the resulting text (i.e., removing punctuation, tags and special characters, and setting all characters to lowercase), (2) a tokenization process in which sentences are split into words, (3) the definition and elimination of a set of stop words from the text (i.e., words commonly occurring in natural language but lacking meaning on their own), and (4) a stemming process to remove suffixes and prefixes [33].

The second step (data transformation) is a process of transforming text data into feature vectors, which can be fed to a classifier [32]. The most common way to do that is by analyzing certain traits of words (commonly their frequency within the text) [32,34]. However, more complex information can be derived from the text using pre-trained neural networks [35].

We tested three different methods for feature extraction to compare the performance of machine learning algorithms based on each set of characteristics:

1. Bag of Words (BOW). In this algorithm, the text is transformed into a group of words from which the frequency, (i.e.,: the number of times a term appears in the text) is calculated [36];
2. Term frequency–inverse document frequency (TFiDF). This algorithm assigns an importance score to each word of the text according to its frequency within the text itself, and within the collection of documents included in the database [37,38];
3. Embedding. This algorithm captures certain types of relationships between words (e.g., morphological, semantic, contextual, syntactic, etc.) computing vector representations of them [39]. More concretely, in this study we used two embedding methods: (1) Word2Vec, which uses a neural network to predict the context of the word, resulting in a feature vector representing said context [40,41], and (2) GloVe, which obtains the vector representation of each word (i.e., target) by factorizing the co-occurrence probability matrix into two smaller matrices: one for the word when it is acting as a context (i.e., the words surrounding the target) and one when it is acting as the target [42].

We applied an oversampling algorithm on the resulting features of the training subset to ensure all classes had the same number of samples. More concretely, we used the nonrandom SVM-based Synthetic Minority Oversampling Technique (SMOTE), which focuses on generating new minority class instances near SVM-defined decision boundaries. The final balanced training set consisted of 1424 samples, with 178 samples per class (i.e., per surgical phase). This number is determined by the maximum number of samples in a phase (in this case corresponding to the normal conversation in the educational OR). Since our model considered the temporality of the input data, we did not apply oversampling to the test dataset.

### 2.3. Classification

In total, four approaches to supervised classification were selected, each based on a different working principle: (1) a linear classifier using logistic regression, (2) a non-linear classifier SVM (3) a classification ensemble learning method based on decision trees (random forest) and (4) an artificial neural network composed of multilayer perceptrons. A 10-fold cross validation was employed on the training dataset to adjust the models’ parameters. Finally, in order to add temporal information, HMMs were coupled after the models with the resulting optimized hyperparameters.

A general overview on each algorithm’s working principles, advantages and disadvantages is given next. Those interested in further exploring these techniques may find more information in the suggested bibliography.

1. Logistic regression. In general, logistic regression measures the relationship between the input features and the classes by estimating the probabilities of belonging to one or another using a logistic function [43]. It is especially useful due to its simplicity and the fact that it provides a probabilistic output (i.e., the probability of the sample belonging in one class), with which we know how confident the prediction is, leading to a wider usage and deeper analysis [43]. However, it needs an exhaustive pre-processing of the data to avoid introducing noisy data to the model, and most importantly, the assumption of linearity can rarely hold (i.e., most data need more complex modelling), thus hindering the model generalization [43]. For our study we trained the classifier using a one-versus-all (OVA) scheme. This scheme involves splitting the multi-class dataset into multiple binary classification problems. A Ridge regression penalty was applied to the loss function of the classifier to account for oversampling.
2. Support vector machines. SVMs seek to classify the input features into two or more classes by means of hyperplanes, such that most of the samples belonging to each class are contained within their corresponding hyperplanes [44]. Ideally, the distance between those hyperplanes would be maximal, to ensure the classes are sufficiently differentiated. Contrary to logistic regression, the hyperplanes created by SVMs are not necessarily linear, which generally improves their performance. SVMs are effective in high dimensional spaces and in those cases where the number of features is much greater than the number of samples [45]. Nevertheless, it is especially prone to errors related to noise (i.e., when one sample can be classified as more than one class) [45]. We trained our SVMs using a radial basis function (RBF) kernel with a unit regularization parameter.
3. Random forests. Decision trees create classification models based on the learning of sequential binary decision rules (branches), which eventually lead to the classification of the input samples [46]. Random forest is a classifier which employs two or more decision trees on different subsets of the training data, and uses the predictions derived from them to create a unique prediction [47]. The main advantage of random forests over other machine learning methods is that they can judge the importance of the feature [48]. On the contrary, random forests have been shown to overfit when encountered with certain noisy classification problems [48]. The main hyperparameter of our model is the criterion, which can either be the entropy (i.e., the minimum necessary information to distinguish classes), or the Gini impurity (i.e., the frequency of the incorrect classification of a random element).
4. Multilayer perceptron (MLP). Neural networks are a set of artificial neurons divided into layers and connected to transmit and share information. Specifically, they are able to extract features and learn from them to create a classification model [49]. In the specific case of this study, an MLP is designed. MLPs are a type of feedforward neural network, in which the information flows from the input layer through the hidden layers (where the model is actually learnt) towards the output layer, where a classification is made, without any cycles or loops between the hidden layers [50]. It is important to maintain a balance between the number of hidden nodes and the time consumed for model training, since the convergence of the weights can be extremely time-consuming when the network is composed of many hidden nodes [51]. On the other hand, an important advantage of MLP is that the coefficients can easily be adapted using the backpropagation algorithm (i.e., the output of the network is compared with the known class to give an indication of how well the network is doing) [52]. This model carries two main hyperparameters to choose from: the activation function (which determines the output of the MLP) and the optimizer (which optimizes the weights). In our case, the activation functions tested are (1) logistic, (2) hyperbolic tangential and (3) rectified linear unit (ReLu). The optimizers were Adam (stochastic gradient) and limited-memory Broyden–Fletcher–Goldfarb–Shanno (LMBFGS, quasi-Newton) [53,54,55,56].
5. Hidden Markov Model. The architecture of an HMM is composed of (1) a set of hidden states, (2) a transition probability matrix (where the elements represent the probability of moving from one hidden state to another), (3) a sequence of observable states, (4) an emission probability matrix (where each element expresses the probability of an observable state being generated from a certain hidden state) and (5) an initial probability distribution over hidden states [57]. The main idea behind using HMMs in classification problems is that they are able to calculate the most statistically probable sequence of hidden states (classes) based on the probability matrices of the observable ones (features). Additionally, they employ sequential and temporal information, which improves the results in the prediction of sequential data (as is our case).

### 2.4. Evaluation

To compare the performance of the different models, F1-scores were calculated against the test set for each of the models and feature extraction techniques (both with and without HMM coupling). The F1-score is the harmonic mean of the precision (number of samples correctly assigned to a phase divided by the number of samples correctly and incorrectly assigned to that phase) and the recall (number of samples correctly assigned to a phase divided by the number of samples correctly assigned to that phase plus the number of samples incorrectly assigned to other phases). In our case it was an appropriate metric to evaluate whether the models correctly classify the surgical phases, since it studies the correct identification of a specific phase without losing sight of the classification of the rest of the phases. In multiclass problems such as this one, F1-scores are given as the average of the F1-score for each class.

On the other hand, we carried out an error analysis by means of (1) analyzing the 10-fold cross-validation results to find signs of over/underfitting, (2) analyzing the model performance when classifying each phase by means of the confusion matrix of the best performing model, and (3) analyzing, for said model, the error rate per phase (i.e., number of incorrectly classified samples per phase) and the average error rate (i.e., number of incorrectly classified samples). This allowed us to find the strongest and weakest points of the best performing model.

Finally, in order to analyze the temporality of the model, a comparison between the actual and the predicted timeline was performed. This will give further insights as to whether the model can be used in real-time surgical–educational environments, and what the model’s main flaws are.

## 3. Results

Table 3 summarizes the average values of F1-scores obtained on the 10-fold cross-validation with the optimal combination of hyperparameters for all possible models. Full hyperparameter combination results can be found in Appendix A. Results suggest that both the random forest classifiers trained with the embedding features (GloVe and Word2Vec) and the MLP trained with Word2Vec features are overfitting. The information from Table 3 suggests that HMM might be improving the models, since the F1-scores obtained for the validation subsets of the cross-validation increase in all cases.

**Figure 3:**
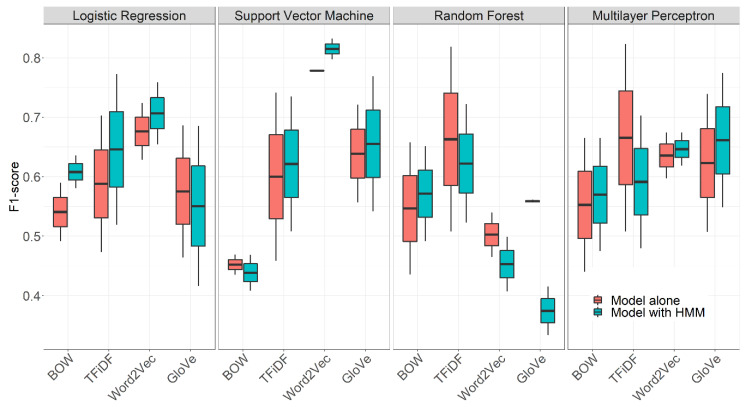
F1-scores obtained in the test subset (two videos) for all features and models.

Figure 3 represents the F1-scores obtained against the test subset for each model and feature extraction technique (both with and without HMM coupling). The model providing the highest F1-score was an SVM coupled with an HMM, outperforming the rest of the models for both videos.

HMM improves the F1-scores with respect to models alone (i.e., with no temporal information), except in the case of random forests. We can also observe that the Word2Vec feature extraction technique provides the best results in most cases, the exception being once more random forests.

**Figure 4:**
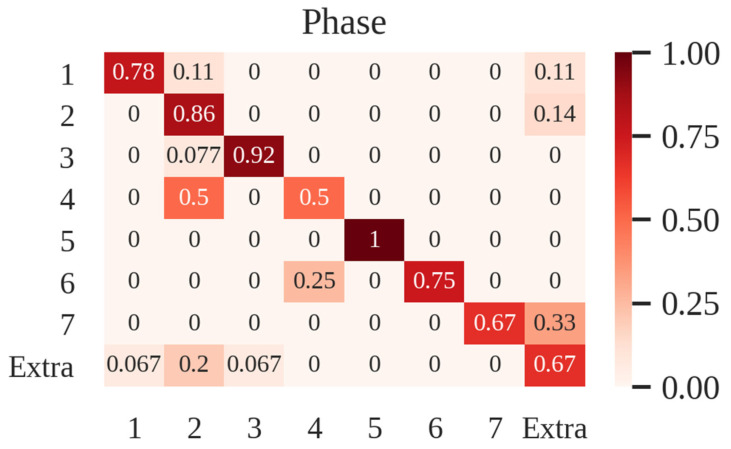
Confusion matrix of the support vector machine–hidden-Markov model (SVM-HMM) for the test dataset.

We computed the average errors per phase (23.08%) and the total percentage of errors (20.69%). The confusion matrix of the best model is shown in Figure 4. Phases 1, 2, 3 and 5 had more than 75% of samples correctly classified. However, phase 4 was easily confused with phase 2 (50%), and 33% of sentences belonging to phase 7 were confused with normal conversation in the OR (“extra” pseudo-phase). Additionally, the samples belonging to this normal conversation were mistakenly classified as phase 2 in a 20% of cases, and as phase 1 and 3 to a lesser extent. Finally, although phase 6 reached 75% of the correctly classified samples, the remaining 25% was confused entirely with phase 4.

**Figure 5:**
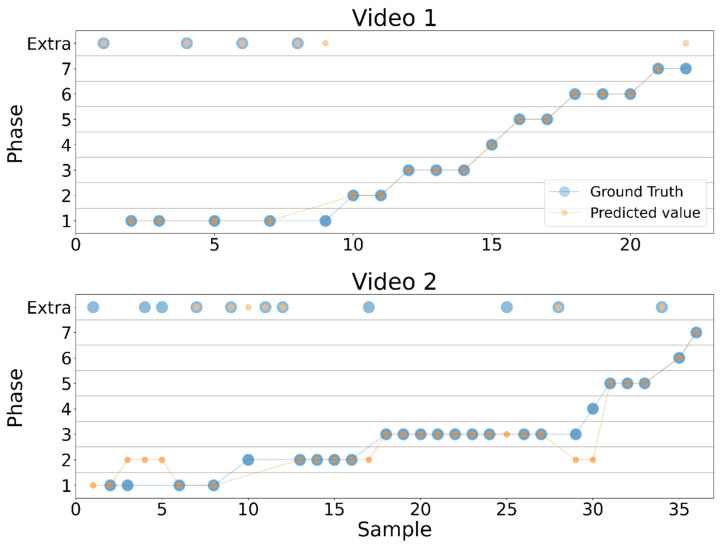
Timeline comparison of the ground truth phases (blue) and the phases predicted by the SVM-HMM model (red) for video 1 and video 2.

Figure 5 shows the timeline comparison between the ground truth phases and those predicted by the SVM-HMM model, corresponding to the samples in sequential order. The classification of the first video is achieved successfully, without making any relevant time-related errors (i.e., the surgery can be followed normally). However, the classification of the second video reveals errors in the classification which disrupt the normal workflow of LC.

## 4. Discussion

In view of the need to develop new non-intrusive methods for monitoring non-technical skills in surgical educational environments, we propose to implement a system to gather the surgeon-in-training speech and analyze it to aid in the assessment of procedural and decision-making skills. The system will recognize the ongoing surgical phases based on the verbal comments of surgical trainees and compare them to the framework of the procedure. To test the feasibility of carrying out phase recognition using speech-based SWA, we have trained four classification models with four different algorithms for feature extraction to find the best approach.

The model with the best results uses Word2Vec to extract features from the text transcriptions and assigns them their corresponding phase by means of an SVM coupled with an HMM, achieving an average accuracy of 82.95% (90.9% for video 1 and 75% for video 2). As mentioned before, one of the main advantages of SVM is their excellent performance in high dimensional spaces [45], as is the case of the features resulting from Word2Vec. The addition of HMM significantly improves the model, since it includes temporal information, which is essential for our particular problem [18].

The fact that Word2Vec is the feature extraction technique yielding the best result is not surprising, since it does not only represent word associations within a sentence, but also detects synonyms [40]. This is a great advantage in our case since surgeons usually employ synonyms to describe the same tasks of the surgical process (e.g., “suturing” and “closing”). However, this plays against random forests due to the high dimensionality of the resulting embedding matrix [47], which may explain why the model based on this technique cannot correctly adjust the data. Additionally, the combination of HMM and embedded random forests performs poorly. This is possibly because the random forest models are not generalizing correctly, due to the small depth of the decision trees conforming their ensemble and the high complexity of the features extracted with both embedding methods. This theory is reinforced by Table 3, in which random forests models seem to be overfitting, for which they were discarded.

Confusion matrix analysis helps to identify the most common errors committed by our algorithm within individual phases, as well as the phases where the accuracy is higher. The best results were obtained for phase 5 (100% accuracy), which features highly characteristic words (e.g., “extraction”, “endobag”) (Figure 4). This makes it more easily differentiable than the rest of the phases. On the other side, phase 4 is confused with phase 2. This might be due to the similarities between the words themselves (i.e., “dissect” is said on both phases). This, together with the fact that Word2Vec obtained the highest F1-scores for both videos, suggests that the context of words should be given higher importance.

It is also worth mentioning the problems related to the pseudo-phase we created for normal conversation in the OR. This “extra” pseudo-phase is confused mainly with phase 2. Even though in real life the words in these two phases may be the same, the context and intention is not the same. What is more, we found great differences between the F1-scores of the two videos in the test subset when training with TFiDF features (Figure 3). TFiDF extracts features based on the frequency of words within the whole database, and we have created a pseudo-phase with words that are difficult to characterize by their frequency (i.e., random). Thus, it seems plausible that TFiDF performs better for the video in which there are fewer pseudo-phase’s samples. This further supports the hypothesis that the context should be given higher importance when extracting features.

Additionally, phase 7 tends to be confused with the “extra” pseudo-phase. A possible explanation for this is that since this phase was the minority class, most of the samples are synthetic, and are more difficult to distinguish. Furthermore, manual inspection of videos revealed that some of them did not properly comment on the suturing and port removal. This may have biased the model for this concrete phase.

The timeline comparison between the annotated phases (ground truth) and the predicted ones overall predicts an appropriate timeline in the first video (Figure 5). On the contrary, the second video contains rather relevant disruptions. More specifically, this video starts in phase 1, goes to phase 2 and then comes back to phase 1, which is highly unlikely in LC, because once dissection has started, all ports are expected to be in their place and there should be no need to go back to that step. In this case, this disruption was caused by misclassifications of normal conversation in the educational OR with phase 2.

In addition to this, the 29th and 30th samples were misclassified as phase 2. Specifically, the 29th sample should have been classified as phase 3 while the 30th sample should have been recognized as phase 4. This causes an important disruption of the workflow because it wrongly assumes that after phase 3, the surgeon goes back to phase 2 and then to phase 5 without any mention of phase 4. This disruption might be motivated by the 28th sample, which corresponds to the pseudo-phase, since the HMM seems to assign higher probability to going from phase 8 to phase 2 than from phase 8 to phase 3. This could be solved by reviewing the transition probabilities of the HMM and applying looser restrictions to go from any phase to the pseudo-phase.

Despite the many algorithms for SWA, current methods are focused on applying probabilistic approaches or deep learning methodologies to extract features and classify video-based data. The speech-based approach could be a useful asset for SWA, providing additional information of the surgical process occurring in the educational OR and potentially linking cognitive processes to the actions undertaken. This is the main advantage of our proposed algorithm. The obtained accuracies from video-based methods analyzed in the literature are within 68.1% and 92%. Thus, the results obtained with our speech-based phase recognition algorithm are within the state-of-the-art (82.95%). Moreover, the only other study identified in the literature to include audio recordings did not analyze surgeons’ speech [23], which speaks of the innovative nature of the proposed method.

Nevertheless, we do acknowledge the difficulties of using speech for phase recognition in surgical educational contexts. The main problem is that the educational OR is extremely noisy, which may pose great challenges when using automatic transcription algorithms to convert speech into text. To solve this issue, a thorough analysis of the main noises in the educational OR must be carried out to identify their sources and how to efficiently remove them.

Even though we obtained a model with state-of-the-art accuracy, we are aware of the limitations of our study. To begin with, the database created for this study contains sentences only in Spanish. Despite Spanish being one of the most spoken languages in the world [58], it would indeed be interesting to see the results of this study extrapolated to other languages (e.g., English). In addition to this, the database could be further optimized (e.g., by including more videos from surgical educational environments, or by having more educational experts annotating the phases).

Another limitation to our work is related to the creation of the database. Although the phases in which we have divided the surgical procedure have been validated [12], the boundaries between each phase may be diffused. This means that some segments could belong to one phase or another indistinctly. We tried to overcome this by including two annotators and correlating their annotations. We also propose to further define these boundaries and create a protocol for annotations to avoid these types of errors in the future.

The greatest challenge in our study was the treatment of the pseudo-phase. Our initial approach was to ignore this type of speech and to train the models only with the relevant samples (i.e., whenever the surgeon-in-training refers to a specific step of the surgery). However, this was not a realistic approach since in a real training environment we would be presented with many verbal statements not necessarily related to a specific phase (e.g., questions or answers to the trainer, conversation with a colleague, etc.). This led us to introduce the pseudo-phase with the aim of categorizing all non-phase-related speech. At this point, the HMM was presented with new challenges that it did not have before: this new pseudo-phase was not part of the surgical sequence but was presented as such. This caused the HMM to generate mistakes around the pseudo-phase, but it improved the classification of the overall model considerably. As part of our efforts to deal with this issue, we propose to use the SVM classifier as a sort of “pseudo-phase” blocker, such that whenever normal conversation is recognized by the SVM, it does not go through the HMM.

To further validate the performance of the model, it is necessary to increase the accuracy of the models alone, for example by including deep learning algorithms. This will require a database with a higher number of samples. In fact, we acknowledge this is one of our work’s limitations and it is our priority for future studies, where we aim to gather new custom-made audio recordings at surgical training centers, from which we can effectively extract the speech of the surgeon-in-training.

The new data acquisition process would be carried out according to the general data protection regulation (GDPR) and would require consent from all participants, to deal with the issues with data protection and privacy rights. We are aware that these issues need to be addressed with caution to ensure the audio recorded is only used for the classification of surgical phases. For instance, the data could be analyzed while it is being captured by the acquisition system instead of saved on disk. This type of real-time analysis requires extra care in the audio pre-processing step (e.g., the noise of the audio signals should be completely removed, the precision of the algorithm to divide speech data into sentences according to the silences of the main surgeon should be extremely accurate, etc.).

All things considered, the results lay the foundations for speech-based SWA, showing it is indeed possible to recognize surgical phases. However, since we have manually transcribed all the audio in the database, the next step will be an analysis of automatic transcribers’ methods for the specific context of surgical education. More specifically, transcribers will be used to automatically transform the audio signals obtained from the videos conforming the database used for this study into text. The results from each transcriber will be compared with the actual transcription to find the most effective transcriber for our specific task (taking into account the noise present in surgical-educational environments). This is a key step for the recognition of surgical phases in real time.

We also aim to improve the chunking algorithm, to account for the fact that people may explain more than one idea before pausing (i.e., whenever a silence is detected). For instance, we could incorporate a time limitation to the chunking process to ensure that a sentence is not too long and includes too many ideas. A more complex hypothesis would be to divide the audio whenever a certain word was said by the speaker (e.g., “cut”, “dissect”). This indeed requires a thorough understanding of the cognitive process behind the surgical procedure.

Regarding the assessment functionality, we aim at testing the validity of the proposed system with surgical residents in the near future. We also plan to study a lower granularity of the procedure. Specifically, we would analyze the main actions in LC and annotate the speech samples accordingly. These new annotations would be used for the training of new models to classify the individual actions within the procedure (instead of the phases), so that we are able to explore the usefulness of this granularity level with respect to the assessment of procedural and decision-making skills.

## 5. Conclusions

This study stems from the need to develop assessment methods for the objective automatic assessment of surgical residents’ cognitive and decision-making skills. To fill this gap, we propose to create a system capable of recognizing surgical phases to create a workflow that can be compared to the framework of the procedure. This will allow to find disruptions within the natural workflow of the procedure, which can be further analyzed as part of the assessment process.

Currently, most methods for phase recognition are based on video analysis, and even though they can be used to accurately classify phases, videos are prone to errors due to camera occlusions and image perturbations. What we propose is to use speech to carry out the classification of phases. Specifically, we propose to do this by means of a system which would filter and transcribe the audio signals and classify the resulting transcriptions into the ongoing surgical phase. It is our belief that speech analysis can ultimately provide invaluable insights in relation to the cognitive processes which guide decision making, and thus may be used as a useful steering tool for surgical learning in the OR.

Based on the results of this study, our next steps will be aimed at the data processing step of the system. More concretely, we will explore new avenues for chunking audio files, as well as delve into the mechanisms of transcribing speech to text. In addition to this, we will focus on improving the accuracy of the current model by (1) exploring the possibility to include a classifier at the first stage to differentiate between random conversation and verbal comments on the surgical procedure, (2) looking into deep learning algorithms to carry out the classification and (3) generating new custom-made data. We will look into the effectiveness of using lower granularity levels (i.e., actions) to carry out the assessment of skills. We will also study the validity of the assessment step of the system by testing it with surgical residents in educational environments.

In conclusion, the results we obtained prove that it is feasible to recognize surgical phases using the surgeon-in-training speech, which is promising in terms of exploring a new paradigm for SWA based on speech analysis.

## Figures and Tables

**Table 1:** Description of the surgical phases of the laparoscopic cholecystectomy.

Description	Start Point	End Point
Phase 1. Preparation.		
Laparoscopic devices are initialized, trocars are placed, and an initial inspection is carried out.	The patient enters the OR.	The endoscope is inside the abdominal cavity.
Phase 2. Calot’s triangle dissection.		
Abdominal adhesions are retracted to correctly recognize the Calot’s triangle.	The anatomical structures are located.	Calot’s triangle anterior and posterior dissection.
Phase 3. Cystic duct and artery clipping and cutting.		
Clips are placed on the cystic duct and artery to avoid bile liberation or bleeding. A cut is made between clips.	The cystic duct and artery are recognized.	The cystic duct and artery have been cut.
Phase 4. Gallbladder dissection.		
The base of the gallbladder is separated from the liver bed, allowing for gallbladder liberation.	The gallbladder is not attached to the cystic duct and artery.	The gallbladder is ready to be removed.
Phase 5. Gallbladder extraction.		
The gallbladder is placed on a bag (“endobag”) and removed from the abdominal cavity.	The gallbladder is separated from the abdominal wall.	The gallbladder is extracted.
Phase 6. Cleaning and coagulation.		
A general inspection is carried out check for bleeding points. Any bleeding is absorbed with drains.	The gallbladder has been extracted.	No blood or damage is detected.
Phase 7. Suturing and port removal.		
Ports are removed and wounds are closed with staples or threads.	The abdominal cavity has been checked for bleeding.	All wounds are closed.

**Table 2:** Summary of the distribution of text samples per phases. (T) indicates the videos used for testing.

Video	Phase 1	Phase 2	Phase 3	Phase 4	Phase 5	Phase 6	Phase 7	Extra	Total
1 (T)	4	5	10	1	3	1	1	11	36
2 (T)	5	2	3	1	2	3	2	4	22
3	9	7	9	2	7	9	2	18	66
4	1	6	6	2	2	1	1	3	22
5	9	5	6	0	1	3	4	16	44
6	0	3	12	4	4	5	0	6	34
7	1	4	5	2	1	5	0	8	26
8	14	28	12	13	14	11	8	61	167
9	2	10	5	6	2	2	0	12	39
10	5	6	3	3	2	0	1	3	23
11	0	2	3	1	1	1	0	4	12
12	0	7	7	5	1	3	0	6	29
13	0	5	6	1	1	1	0	1	15
14	4	6	7	1	2	0	2	7	29
15	5	6	10	0	7	9	1	18	58
Total	59	102	104	42	50	54	22	178	611

**Table 3:** Average value of F1-scores ± standard deviation obtained on the cross-validation with the best hyperparameters.

Model	Subset	BOW	TFiDF	GloVe	Word2Vec
Log	Train	0.91 ± 0.008	0.987 ± 0.002	0.911 ± 0.009	0.997 ± 0.001
	Validation	0.733 ± 0.126	0.841 ± 0.094	0.786 ± 0.089	0.863 ± 0.083
Log + HMM	Train	0.987 ± 0.004	0.995 ± 0.001	0.92 ± 0.009	0.997 ± 0.001
	Validation	0.917 ± 0.074	0.987 ± 0.02	0.89 ± 0.107	0.99 ± 0.016
SVM	Train	0.865 ± 0.007	0.975 ± 0.003	0.935 ± 0.006	0.934 ± 0.004
	Validation	0.704 ± 0.166	0.864 ± 0.104	0.828 ± 0.088	0.836 ± 0.092
SVM + HMM	Train	0.95 ± 0.008	0.98 ± 0.002	0.952 ± 0.007	0.949 ± 0.006
	Validation	0.905 ± 0.083	0.945 ± 0.077	0.927 ± 0.082	0.924 ± 0.086
RF	Train	0.908 ± 0.009	0.99 ± 0.003	1 ± 0	1 ± 0
	Validation	0.696 ± 0.131	0.857 ± 0.11	0.87 ± 0.087	0.884 ± 0.09
RF + HMM	Train	0.95 ± 0.008	0.996 ± 0.001	1 ± 0	0.999 ± 0.003
	Validation	0.906 ± 0.084	0.985 ± 0.023	1 ± 0	0.998 ± 0.006
MLP	Train	0.888 ± 0.01	0.988 ± 0.002	0.848 ± 0.009	1 ± 0
	Validation	0.696 ± 0.132	0.849 ± 0.092	0.76 ± 0.105	0.868 ± 0.084
MLP + HMM	Train	0.987 ± 0.002	0.998 ± 0.001	0.892 ± 0.02	0.999 ± 0.001
	Validation	0.918 ± 0.143	0.99 ± 0.02	0.797 ± 0.3	1 ± 0

## Data Availability

Data available on request.

## Appendix A

**Table A1:** Hyperparameters of the ML optimized models.

LOGISTIC REGRESSION							
	**C**	**Class weight**	**Solver**	**Tolerance**		**Warm start**	
BOW	100	Weighted	Liblinear	0.0001		TRUE	
TFiDF	100	Weighted	Saga	1.00× 10^−5^		TRUE	
GloVe	100	Not weighted	Newton-cg	0.0001		TRUE	
Word2Vec	100	Weighted	Sag	1.00× 10^−5^		FALSE	
SUPPORT VECTOR MACHINE							
	**Decision function shape**		**Degree**		**Tolerance**		
BOW	OVO		1		0.01		
TFiDF	OVO		1		0.01		
GloVe	OVO		1		0.01		
Word2Vec	OVO		1		0.001		
RANDOM FOREST							
	**Criterion**	**Min. #samples to split internal node**		**Number of estimators**		**OOB score**	
BOW	Gini	4		25		TRUE	
TFiDF	Entropy	4		50		FALSE	
GloVe	Entropy	2		500		FALSE	
Word2Vec	Entropy	2		500		FALSE	
MULTILAYER PERCEPTRON							
	**Activation**	**Hidden layer size**	**Learning rate**	**Shuffle**	**Tolerance**		**Solver**
BOW	Relu	100	Invscale	TRUE	1.00× 10^−6^		Adam
TFiDF	Relu	100	Adapive	TRUE	0.0001		Adam
GloVe	Tanh	100	Constante	FALSE	1.00× 10^−5^		lbfgs
Word2Vec	Logistic	100	Invscale	TRUE	1.00× 10^−5^		lbfgs
